# Supplementary material for: Shape-changing electrode array for minimally invasive large-scale intracranial brain activity mapping
Source: Nat Commun. 2024 Jan 24;15:715. doi: 10.1038/s41467-024-44805-2 (PMC10808108; doi:10.1038/s41467-024-44805-2)
Supplement: Supplementary file 1 — Supplementary Information [file 41467_2024_44805_MOESM1_ESM.pdf]

Supplementary Information: Shape-changing electrode array for minimally invasive large-scale intracranial brain activity mapping

Shiyuan Wei[1,2]†, Anqi Jiang[1]†, Hongji Sun[1], Jingjun Zhu[1,3], Shengyi Jia[1], Xiaojun Liu[1], Zheng Xu[1], Jing Zhang[1,2], Yuanyuan Shang[4], Xuefeng Fu[1], Gen Li[1], Puxin Wang[1,2], Zhiyuan Xia[5], Tianzi Jiang[6], Anyuan Cao[5], Xiaojie Duan[1,2,3]*

[1] Department of Biomedical Engineering, College of Future Technology, Peking University, Beijing 100871, China.

[2] Academy for Advanced Interdisciplinary Studies, Peking University, Beijing 100871, China.

[3] National Biomedical Imaging Centre, Peking University, Beijing 100871, China.

[4] Key Laboratory of Material Physics, Ministry of Education, School of Physics and Microelectronics, Zhengzhou University, Zhengzhou 450052, China.

[5] School of Materials Science and Engineering, Peking University, Beijing, China.

[6] Brainnetome Centre, Institute of Automation, Chinese Academy of Sciences (CAS), Beijing 100190, China.

† These authors contributed equally to this work.

*Correspondence and requests for materials should be addressed to X. D. (email: xjduan@pku.edu.cn)

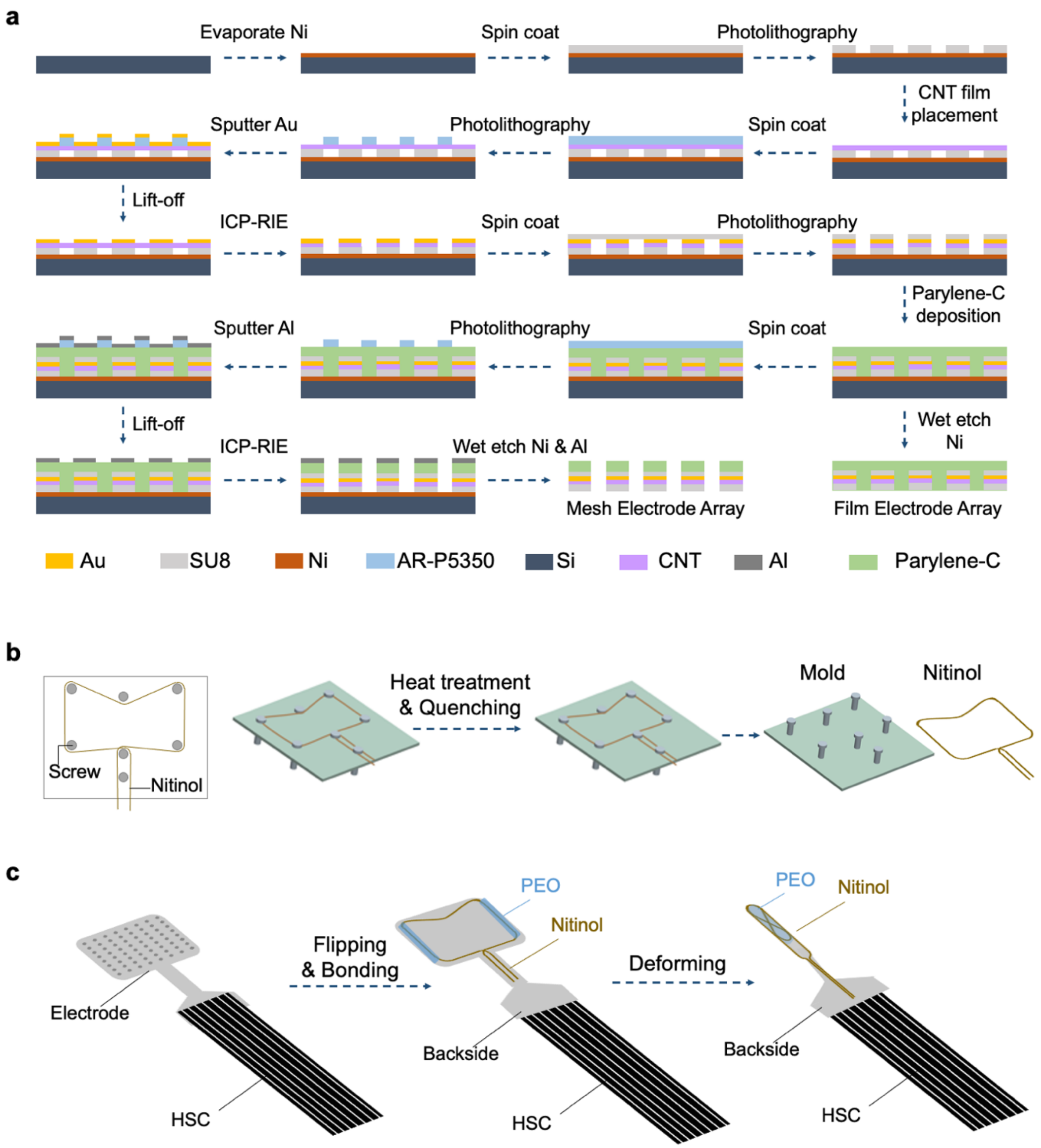

Fig. S1 | Fabrication of a SCEA. a, Schematics showing the fabrication steps of the CNT/Au electrode array. **b**, Schematics showing the method used for nitinol actuator shaping. **c**, Schematics showing the bonding and compression of the CNT/Au array and nitinol complex. The nitinol shape actuator was attached to the backside of the CNT/Au array using polyethylene oxide (PEO)

solution, and remained on the backside of the array throughout the deformation process, as depicted in the figure.

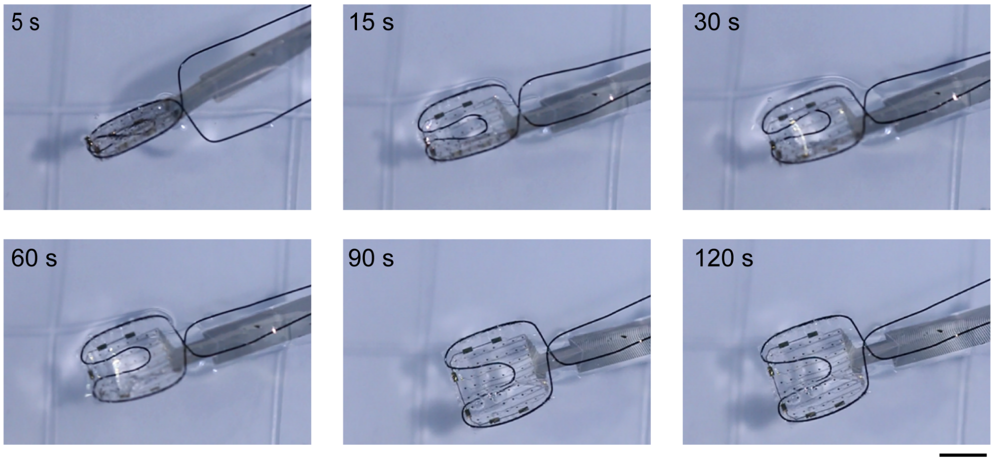

Fig. S2 | Deployment of a SCEA *in vitro*. The SCEA was placed on the surface of an agar gel surrounded by 37°C saline. Scale bar, 3 mm.

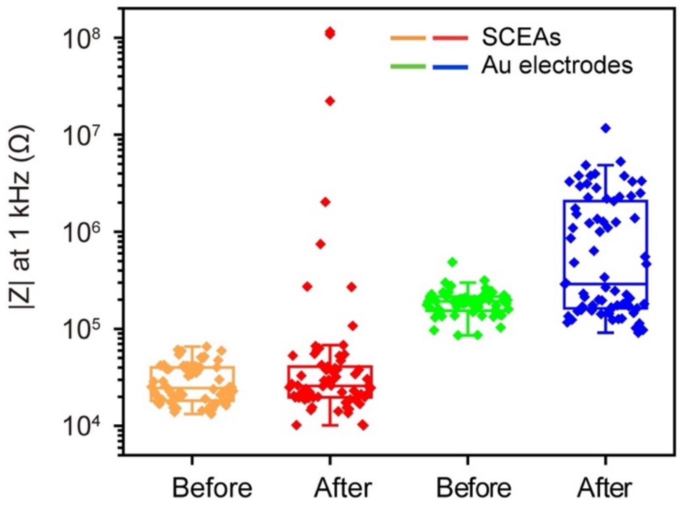

Fig. S3 | Comparison between SCEAs and pure Au electrode array on impedance change after one shape transformation cycle (sheet to strip, and then back to sheet). Both types of electrodes had circular active sites with a diameter of 250 μm. The box plots show the median and quartile range, and the whiskers denote 1.5× the interquartile range. n=72 from 5 Au electrodes, n=72 from 5 CNT SCEAs.

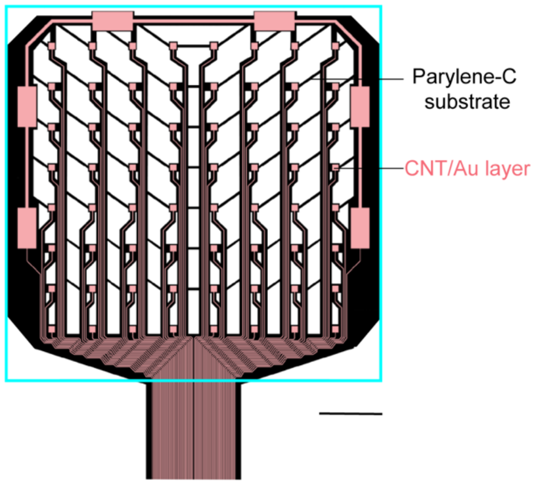

Fig. S4 | Mesh SCEA design. The pattern of CNT/Au layer and Parylene substrate is shown in pink and black, respectively. Scale bar, 1 mm.

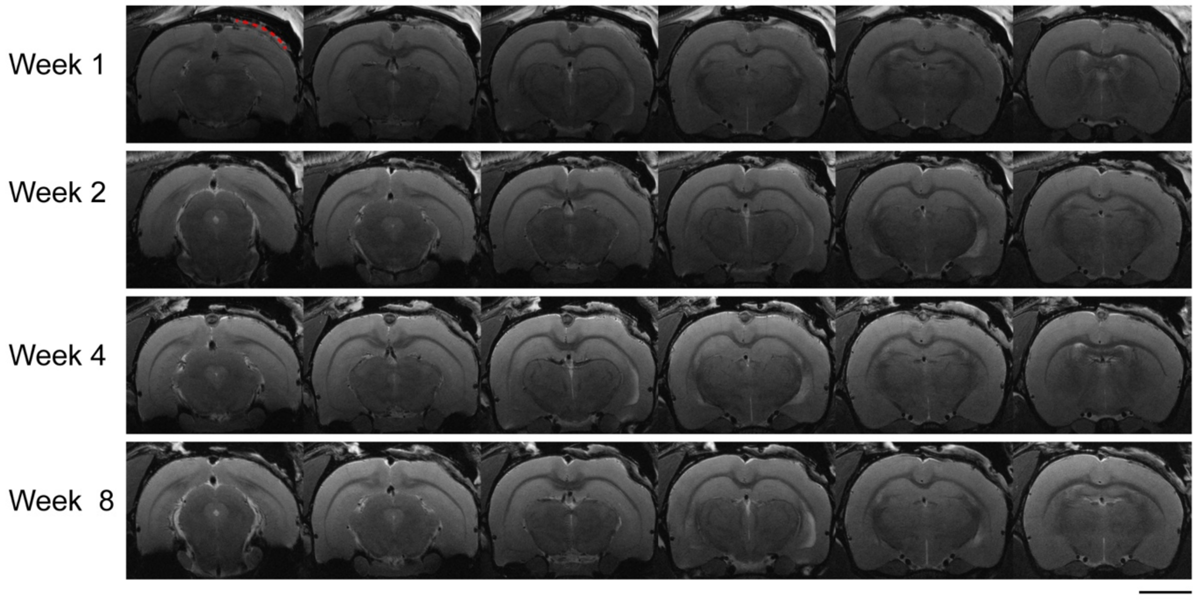

Fig. S5 | Representative T2-weighted MRI images of a rat brain at different time points after the implantation of a mesh CNT/Au electrode array through conventional craniotomy. Images were acquired at 1, 2, 4 and 8 weeks postimplantation. The red dotted line indicates the position of the mesh CNT/Au electrode array. Scale bar, 5 mm.

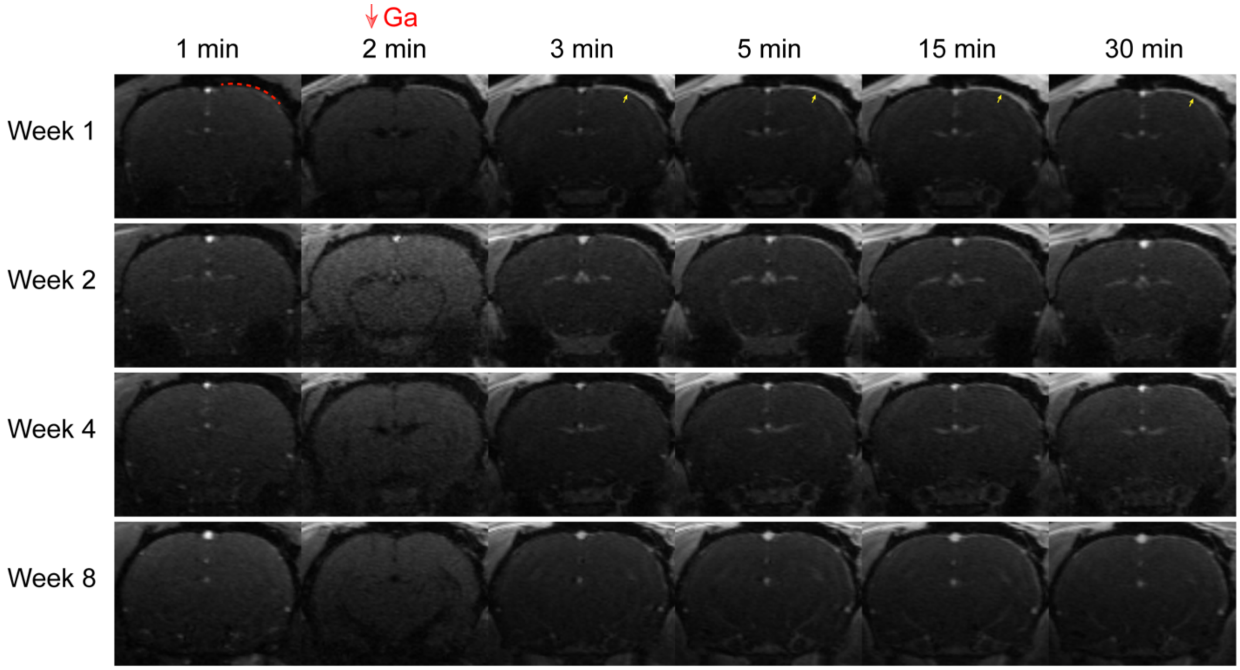

Fig. S6 | Representative DCE-MRI images of a rat brain at different time points after the implantation of a mesh CNT/Au electrode array through conventional craniotomy. The time on top of the MRI images shows the time when the MRI images were acquired in each measurement. Baseline T2 images were acquired at 1 min. The bolus injection of the CA at 2 min was associated with a reduction in signal intensity throughout the brain. The red dotted line shows the position of the CNT/Au mesh array. The yellow arrows point to locations with meningeal enhancement. Scale bar, 5 mm.

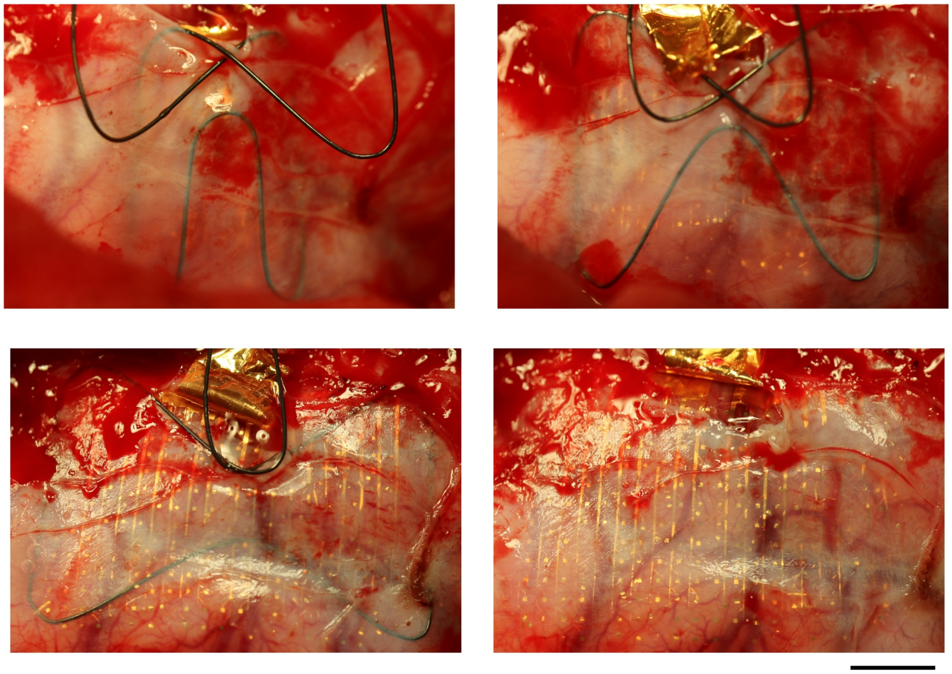

Fig. S7 | Deployment of a SCEA under the dura mater in a beagle brain. The pictures were taken at different time points during the deployment. Scale bar, 5 mm.

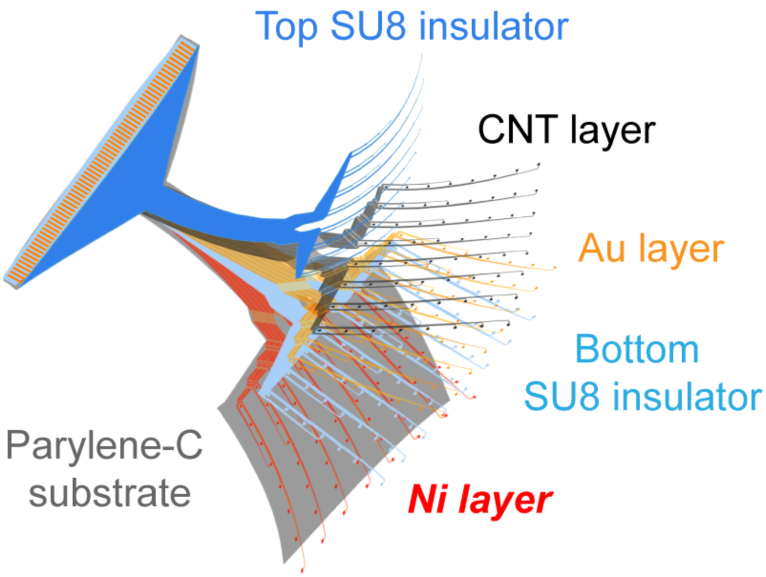

Fig. S8 | Expanded schematic view of the layered structure of the Ni-SCEA. The nickel layer was placed between the bottom SU8 layer and the Parylene substrate with the same pattern as the CNT/Au layers and with a thickness of 100 nm.
