# Supplementary material for: Shape-changing electrode array for minimally invasive large-scale intracranial brain activity mapping
Source: Nat Commun. 2024 Jan 24;15:715. doi: 10.1038/s41467-024-44805-2 (PMC10808108; doi:10.1038/s41467-024-44805-2)
Supplement: Supplementary file 2 — Description of Additional Supplementary Files [file 41467_2024_44805_MOESM2_ESM.pdf]

Description of Additional Supplementary Files

File name: Supplementary Movie 1

Description: Deployment of a SCEA in vitro.

File name: Supplementary Movie 2

Description: Implantation of a SCEA in a rat epidurally. The skull of the rat was thinned by drilling for visualization. The video showcases the insertion of the SCEA strip, the expansion of the nitinol and SCEA beneath the skull, the retrieval of the nitinol actuator, and the subsequent removal of the electrode array from the brain surface through the cranial opening. The video playback speed is 8x. The entire process took approximately 45 minutes. The segment from 2 min 16 s to 40 min displays the implanted device remaining on the brain surface for PEO dissolution and actuator-electrode array detachment, during which no further operation was conducted. To maintain a reasonable video size, this unchanging section was omitted, while the preceding and following phases were labeled as Supplementary Movie 2 and Supplementary Movie 3, respectively.

File name: Supplementary Movie 3

Description: Implantation of a SCEA in a rat epidurally. The skull of the rat was thinned by drilling for visualization. The video showcases the insertion of the SCEA strip, the expansion of the nitinol and SCEA beneath the skull, the retrieval of the nitinol actuator, and the subsequent removal of the electrode array from the brain surface through the cranial opening. The video playback speed is 8x. The entire process took approximately 45 minutes. The segment from 2 min 16 s to 40 min displays the implanted device remaining on the brain surface for PEO dissolution and actuator-electrode array detachment, during which no further operation was conducted. To maintain a reasonable video size, this unchanging section was omitted, while the preceding and following phases were labeled as Supplementary Movie 2 and Supplementary Movie 3, respectively.
