# Supplementary material for: Shape-changing electrode array for minimally invasive large-scale intracranial brain activity mapping
Source: Nat Commun. 2024 Jan 24;15:715. doi: 10.1038/s41467-024-44805-2 (PMC10808108; doi:10.1038/s41467-024-44805-2)
Supplement: Supplementary file 6 — Reporting Summary [file 41467_2024_44805_MOESM6_ESM.pdf]

nature portfolio

Corresponding author(s): Xiaojie Duan

Last updated by author(s): Nov 22, 2023

Reporting Summary

Nature Portfolio wishes to improve the reproducibility of the work that we publish. This form provides structure for consistency and transparency in reporting. For further information on Nature Portfolio policies, see our Editorial Policies and the Editorial Policy Checklist.

Statistics

For all statistical analyses, confirm that the following items are present in the figure legend, table legend, main text, or Methods section.

n/a	Confirmed	
☐	☒	The exact sample size (*n*) for each experimental group/condition, given as a discrete number and unit of measurement
☐	☒	A statement on whether measurements were taken from distinct samples or whether the same sample was measured repeatedly
☐	☒	The statistical test(s) used AND whether they are one- or two-sided<br>*Only common tests should be described solely by name; describe more complex techniques in the Methods section.*
☐	☒	A description of all covariates tested
☐	☒	A description of any assumptions or corrections, such as tests of normality and adjustment for multiple comparisons
☐	☒	A full description of the statistical parameters including central tendency (e.g. means) or other basic estimates (e.g. regression coefficient) AND variation (e.g. standard deviation) or associated estimates of uncertainty (e.g. confidence intervals)
☐	☒	For null hypothesis testing, the test statistic (e.g. F, t, r) with confidence intervals, effect sizes, degrees of freedom and P value noted<br>*Give P values as exact values whenever suitable.*
☒	☐	For Bayesian analysis, information on the choice of priors and Markov chain Monte Carlo settings
☐	☒	For hierarchical and complex designs, identification of the appropriate level for tests and full reporting of outcomes
☒	☐	Estimates of effect sizes (e.g. Cohen's *d*, Pearson's *r*), indicating how they were calculated

Our web collection on statistics for biologists contains articles on many of the points above.

Software and code

Policy information about availability of computer code

Data collection	CHI660E Electrochemical workstation version 15.08 for electrochemical data collection; ParaVision Version 6.0.1 was used for MRI acquisitions. Confocal imaging was performed on a DIVE system (TCS SP8 DIVE, Leica, Germany). Electrophysiological data was collected by Intan preamplifier system (RHD2164, Intan Technologies LLC., USA).
Data analysis	Origin 2020 for plotting data and data analysis; Matlab2022b for data analysis and plotting data; matlab chronux toolbox was used for coherence calculations; ImageJ for MRI and fluorescence image analysis; Neuroexplorer version 5 software for data analysis;MIPAV and itk-SNAP software were used for MRI image analysis. All the codes used for analysis have been provided on the Open Science Framework under ID: kucgw (https://osf.io/kucgw).

For manuscripts utilizing custom algorithms or software that are central to the research but not yet described in published literature, software must be made available to editors and reviewers. We strongly encourage code deposition in a community repository (e.g. GitHub). See the Nature Portfolio guidelines for submitting code & software for further information.

Data

Policy information about availability of data

All manuscripts must include a data availability statement. This statement should provide the following information, where applicable:

- Accession codes, unique identifiers, or web links for publicly available datasets
- A description of any restrictions on data availability
- For clinical datasets or third party data, please ensure that the statement adheres to our policy

Source data have been provided in the Source Data. xls file. All the raw data for MRI, CT and fluorescent images have been uploaded to the Open Science Framework under ID: kucgw.(https://osf.io/kucgw).

Human research participants

Policy information about studies involving human research participants and Sex and Gender in Research.

Reporting on sex and gender	N/A
Population characteristics	N/A
Recruitment	N/A
Ethics oversight	N/A

Note that full information on the approval of the study protocol must also be provided in the manuscript.

Field-specific reporting

Please select the one below that is the best fit for your research. If you are not sure, read the appropriate sections before making your selection.

☒ Life sciences ☐ Behavioural & social sciences ☐ Ecological, evolutionary & environmental sciences

For a reference copy of the document with all sections, see nature.com/documents/nr-reporting-summary-flat.pdf

Life sciences study design

All studies must disclose on these points even when the disclosure is negative.

Sample size	sample size was estimated based on previous similar studies. [Yang, Xiao, et al. "Bioinspired neuron-like electronics." Nature materials 18.5 (2019): 510-517.]
Data exclusions	For the immuhistological study, the blurred imaging would be excluded because of the insufficient quality for analysis.
Replication	The number of repetitions for each experiment has been indicated in the manuscript. Attempts at replication were successful and the conclusion were drawn from the analysis of multiple experiments.
Randomization	The animals were randomizedly chosen for implantation of electrode. The electrode was implanted to one hemisphere as experimental group, the other hemisphere of the same animal is served as control group.
Blinding	Investigators were blinded to data collection and analyses.

Reporting for specific materials, systems and methods

We require information from authors about some types of materials, experimental systems and methods used in many studies. Here, indicate whether each material, system or method listed is relevant to your study. If you are not sure if a list item applies to your research, read the appropriate section before selecting a response.

Materials & experimental systems

n/a	Involved in the study
☐	☒ Antibodies
☒	☐ Eukaryotic cell lines
☒	☐ Palaeontology and archaeology
☐	☒ Animals and other organisms
☒	☐ Clinical data
☒	☐ Dual use research of concern

Methods

n/a	Involved in the study
☒	☐ ChIP-seq
☒	☐ Flow cytometry
☐	☒ MRI-based neuroimaging

Antibodies

Antibodies used	chicken anti-glial fibrillary acidic protein (GFAP) (targeting astrocytes, 1:1000, Abcam #ab4674, USA), goat anti-ionized calcium binding adaptor molecule 1 (Iba 1) (targeting microglia, 1:500, Abcam #ab5076, USA), and rabbit anti-neuronal nuclear (NeuN) (targeting nuclei of neurons, 1:1000, Abcam #ab177487, USA). Alexa Fluor 647 donkey anti-rabbit (1:500, Abcam #ab150075, USA); Alexa Fluor 568 donkey anti-goat (1:500, Abcam #A11057, USA); Alexa Fluor 488 donkey anti-chicken (1:500, Abcam #703-545-155, USA).
Validation	chicken anti-glial fibrillary acidic protein (GFAP) (Abcam #ab4674) reacts with mouse and rats;<br>goat anti-ionized calcium binding adaptor molecule 1 (Iba 1) (Abcam #ab5076) reacts with rats and human;<br>rabbit anti-neuronal nuclear (NeuN) (Abcam #ab177487) reacts with mouse, rat, sheep, goat, cat, dog, human, zebrafish,common marmost.<br>Validation statement on the manufacturer's website<br>https://www.abcam.com/gfap-antibody-ab4674.html<br>https://www.abcam.com/neun-antibody-epr12763-neuronal-marker-ab177487.html<br>https://www.abcam.com/iba1-antibody-ab5076.html

Animals and other research organisms

Policy information about studies involving animals; ARRIVE guidelines recommended for reporting animal research, and Sex and Gender in Research

Laboratory animals	Adult Sprague Dawley male rats (6-12 weeks, Charles River Laboratories Inc., China) weighing 30-400 g were used throughout this study. Rats were housed at a temperature is 22 ± 1 degree Celsius and humidity is 30-70%. The adult male beagles were obtained from and housed at a temperature is 22 ± 1 degree Celsius and humidity is 30-70% at Beijing Sincgene company, China.
Wild animals	The study did not involve wild animals.
Reporting on sex	Sex is not considered in this study
Field-collected samples	The study did not involve samples collected from the field.
Ethics oversight	Animal experiments were conducted complying with Beijing Administration Rules of Laboratory Animals and the National Standards of Laboratory Animal Requirements of Environment and Housing Facilities (GB 14925-2010). The rodent experiments were approved by the Institutional Animal Care and Use Committees of Peking University (#COE-DuanXJ-1).<br>Canine experiments were approved by Sincgene Institutional Animal Care and Use Committee (XNG-IAC-20210401).

Note that full information on the approval of the study protocol must also be provided in the manuscript.

Magnetic resonance imaging

Experimental design

Design type	resting state
Design specifications	The number of trials is 1 in each animal per session; For the DCE-MRI, the length of the each trial is 30 minutes.
Behavioral performance measures	The study did not involve behavioral performance measure

Acquisition

Imaging type(s)	structural and perfusion
Field strength	9.4T for rats and 3T for beagles
Sequence & imaging parameters	T2-weighted anatomical images were acquired with parameters as follows: repetition time (TR) /echo time (TE) =2200/33 ms, RARE factor = 8, field-of-view (FOV) = 25*23 mm2, matrix size = 512 * 512, and slice thickness = 0.7 mm.

DCE-MRI images were acquired using FLASH sequence with parameters as follows: TR/TE = 15.625/1.7 ms, flip angle = 18, FOV = 25*23 mm2, matrix size = 128*128, and slice thickness = 0.8 mm.

Area of acquisition: whole brain

Diffusion MRI: ☐ Used ☒ Not used

Preprocessing

Preprocessing software	For MRI imaging of beagles, skull stripping was done by the Medical Image Processing, Analysis and Visualization MIPAV software (http://www.mipav.cit.nih.gov).
Normalization	For MRI imaging of beagles, the subject's structural images were registrated to the atlas structural images of population template ; linear transformation.
Normalization template	Beagle dog structural images of population template (https://ecommons.cornell.edu/handle/1813/67018).
Noise and artifact removal	This study did not involve noise and artifact removal.
Volume censoring	This study did not involve volume censoring.

Statistical modeling & inference

Model type and settings	*Specify type (mass univariate, multivariate, RSA, predictive, etc.) and describe essential details of the model at the first and second levels (e.g. fixed, random or mixed effects; drift or auto-correlation).*
Effect(s) tested	*Define precise effect in terms of the task or stimulus conditions instead of psychological concepts and indicate whether ANOVA or factorial designs were used.*

Specify type of analysis: ☐ Whole brain ☐ ROI-based ☐ Both

Statistic type for inference (See Eklund et al. 2016)	*Specify voxel-wise or cluster-wise and report all relevant parameters for cluster-wise methods.*
Correction	*Describe the type of correction and how it is obtained for multiple comparisons (e.g. FWE, FDR, permutation or Monte Carlo).*

Models & analysis

n/a	Involved in the study
☒	☐ Functional and/or effective connectivity
☐	☒ Graph analysis
☒	☐ Multivariate modeling or predictive analysis

Graph analysis: For DCE-MRI analysis, to quantify the enhancement of cortical signal intensity, the ratio of the mean gray values in the regions of interests, were calculated at each time point as the indicator for cortical BBB breach.
